# Time to first sexual experience and its predictors among young women (15–24 years) in Zambia: Evidence from 2024 Demographic and Health Survey

**DOI:** 10.1371/journal.pgph.0006687

**Published:** 2026-06-22

**Authors:** Emmanuel Musonda, Peter Mumba, Robert Zulu, Sibongile Namayawa, Milika Sikaluzwe, Bwalya Bupe Bwalya, Million Phiri

**Affiliations:** 1 Department of Demography, Population Sciences, Monitoring and Evaluation, School of Humanities and Social Sciences, University of Zambia, Lusaka, Zambia; 2 Department of Economics, School of Social Science, Mulungushi University, Kabwe, Zambia; 3 Centre for Social Development, School of Social Sciences, University of Johannesburg, Johannesburg, South Africa; University of Nigeria - Enugu Campus, NIGERIA

## Abstract

Early sexual experience among young women increases the risk of unintended pregnancies, sexually transmitted infections, and adverse psychosocial and health outcomes. Examining the timing to first sexual experience is very vital in informing sexual and reproductive health policies and interventions aimed at reducing the health risks among young women. However, there is limited recent evidence in Zambia, particularly studies utilizing nationally representative data such as the 2024 Zambia Demographic and Health Survey. Therefore, the study aimed to investigate the determinants of time to first sexual experience among young women in Zambia. Secondary data from the Zambia Demographic and Health Survey (ZDHS 2024) was used in the study. A weighted sample of 5,833 young women was considered in the study. Kaplan–Meier curve, log-rank test, and Cox proportional hazards regression were used to establish the median age at first sexual experience and identify determinants of time to first sexual experience, with statistical significance set at p < 0.05. The study found that 70% (95% CI: 0.71 – 0.73) of the young women had their first sexual experience before the age of 25. The overal median age to first sexual experience was 17 years. Multivariable Cox regression results showed that lower hazards of earlier sexual experience were associated with being aged 20–24 years (AHR = 0.75, 95% CI: 0.70–0.80), coming from a rich household (AHR = 0.75, 95% CI: 0.67–0.85), and being literate (AHR = 0.87, 95% CI: 0.80–0.94). The findings show that age, household wealth, and literacy are significant factors associated with the timing of first sexual experience among young women in Zambia. Interventions to delay sexual initiation should focus on improving girls’ education and addressing socioeconomic inequalities.

## Introduction

The time to first sexual experience among young women continues to attract significant interest, it marks the onset of exposure to adverse sexual and reproductive health outcomes. This transition carries serious implications that can negatively influence future sexual behaviour and health outcomes. Some of the major consequences of early sexual experience include unintended pregnancies and sexually transmitted infections (STIs) [1–3]. For example, in 2020, the global prevalence of STIs among adolescents and young people was estimated at 4% [4]. In Sub-Saharan Africa (SSA), the prevalence was slightly higher, at 6.9%, among young people reporting an STI or genital discharge [5,6]. In Zambia, the prevalence of human immunodeficiency virus (HIV) among adolescents and young people aged 15–24 was estimated at 3.8% [7]. In addition, teenage pregnancy remains a major concern in SSA, with an estimated 18% of adolescents affected [8]. Furthermore, in Zambia, 20.9% of girls become pregnant before the age of 18 [9]. Recent evidence shows that although the average age at first sexual experience in SSA is gradually rising, a considerable proportion of adolescents still engage in sexual activity before the age of 15 [10]. For instance, a study conducted in Rwanda using secondary data from Rwanda Demographic and Health Survey (RDHS) showed that the median age at first sexual experience was 16 years [10].

Several studies have established a strong link between the timing of sexual experience and a wide range of socio-demographic, economic, and behavioural factors. Variables such as age, education, religion, place of residence, region, marital status, frequency of listening to the radio, wealth index, literacy, employment, and exposure to mass media have all been found to influence the timing of first sexual experience [11–13]. Studies conducted in Nigeria, Tanzania and Uganda found that increasing age, living in rural areas, having tertiary education, being married, and owning a phone were associated with a lower risk of early sexual experience [12,14,15]. When factors are identified and comprehended, this will influence policymakers and healthcare providers to develop strategies to promote healthy behaviour and it will also act as an empowerment for women to make informed decision about their sexual health [16].

According to the Zambia National Adolescent Health Strategic Plan (2022–2026), one of the key interventions is to strengthen community education and health promotion on HIV and STI prevention, care, and treatment among adolescents and young people, with a particular focus on delaying sexual experience. This policy recognizes that postponing sexual initiation is a crucial strategy for reducing the prevalence of STIs and early pregnancies. However, while the priority is well established at the policy level, there remains a gap in evidence regarding the timing of sexual experience and the factors that influence it among young women in Zambia. [17].

Despite notable progress in reproductive health research, there remains a significant gap in understanding the timing of first sexual experience among young women in Zambia. While existing studies have largely focused on issues such as HIV testing, STIs and teenage pregnancies, limited attention has been given to the factors that influence when young women initiate sexual activity [18–20]. Therefore, there is limited empirical evidence on the underlying socioeconomic, demographic, and informational drivers that shape when young women initiate sexual activity, particularly within the Zambian context where cultural norms, gender dynamics, and access to reproductive health information vary significantly across settings. This gap is especially important because Zambia continues to experience relatively high rates of adolescent fertility and STI burden, suggesting that existing interventions may not sufficiently address upstream determinants such as the timing to first sexual experience. By explicitly examining the factors influencing sexual experience, this study moves beyond descriptive outcomes to provide context-specific insights that can inform targeted, prevention-oriented interventions. In doing so, it advances existing research by positioning first sexual experience not merely as a correlate of reproductive health outcomes, but as a critical entry point for understanding and mitigating risk among young women in Zambia [17]. Therefore, this study seeks to address this gap by exploring the factors associated with the timing to first sexual experience among this population.

### Theoretical framework

This study is guided by the Health Belief Model (HBM), one of the earliest and most widely used psychological models for explaining and predicting health-related behaviours [21]. The HBM suggests that individuals are more likely to engage in preventive health actions when they perceive themselves to be at risk of a negative health outcome and believe that taking a particular action will reduce that risk. In the context of the timing to first sexual experience, the model provides a useful perspective for understanding how young women’s perceptions and beliefs influence the timing of first sexual experience [22,23].

The HBM emphasizes several key constructs: perceived susceptibility (an individual’s assessment of their risk of experiencing adverse outcomes such as unintended pregnancy or sexually transmitted infections), perceived severity (the seriousness attributed to these outcomes, including both health and social consequences), perceived benefits (belief in the effectiveness of delaying sexual initiation), and perceived barriers (factors that hinder preventive behaviour). Additional components include external or internal triggers such as peer influence, media exposure, or educational messages and self-efficacy (confidence in one’s ability to delay sexual initiation or practice self-control) [22,24,25].

Applying the HBM to this study allows for a more systematic understanding of how socioeconomic, demographic, and informational factors shape the timing to first sexual experience among young women aged 15–24 years in Zambia. Socioeconomic and demographic characteristics such as age, education, household wealth, and place of residence can influence individual perceptions of risk and access to information. Informational factors, including exposure to sexual and reproductive health education and media, act as a trigger that may either encourage or delay sexual activity initiation. These factors interact to shape perceived susceptibility, perceived severity, and self-efficacy, ultimately influencing behavioural outcomes [24,26,27].

Conceptually, the framework assumes that factors such as socioeconomic and demographic characteristics and proximal factors (such as access to information and knowledge) jointly influence individual perceptions as outlined in the HBM. These perceptions, in turn, determine the likelihood and timing of first sexual experience. By integrating these elements, the study moves beyond a purely descriptive analysis and provides a theoretically grounded explanation of the pathways through which different factors influence sexual initiation. This theoretical framing strengthens the analysis by linking observed variables to established behavioural contexts, thereby offering a more robust explanation of the determinants of the timing to first sexual experience [22,28,29].

## Methods and data

### Ethics statement

Permission to access the datasets for Zambia was requested from the DHS Program. Since all ethical protocols were adhered to by ICF International and the respective national statistical agencies during the original data collection process, no additional ethical approval was required for the secondary analysis (DHS Program 2021). The dataset that was analysed is publicly available and may be found at the website (https://dhsprogram.com/).

### Data source

Data from the Zambia Demographic and Health Survey (ZDHS- 2024) was used in this study. Specifically, the study used the women’s file (IR) which contains the responses of women aged 15–49 years. The Demographic and Health Survey (DHS) is a nationwide survey that is carried out across low-and middle-income countries every five-years [30] and collects data on a number of indicators such as marriage, sexual-activity, fertility, fertility-preferences and family-planning [30]. The data were retrieved from the DHS program official database website (http://dhsprogram.com/) for the survey conducted in Zambia. To conduct the 2024 ZDHS, the 2022 Census of Population and Housing (CPH) was used as the sampling frame. Zambia's administrative units included provinces, districts, constituencies, wards, census supervisory areas (CSAs), and enumeration areas (EAs). EAs, averaging 111 households each, were used as the sampling units. The EA frame was updated to reflect changes up to 2017. It included information on households, population counts, and boundary maps”.

The ZDHS employed a two-stage sampling design. In the first stage, 545 clusters were selected from enumeration areas (EAs), proportionate to their size within each stratum. In the second stage, households were systematically sampled within the selected clusters. A total of 13,625 households were selected, with an average of 25 households per cluster. The survey results are representative at the national, urban, rural, and provincial levels. Eligible respondents included women aged 15–49 years who were either permanent residents of the selected households or visitors who stayed overnight before the survey. This analysis was based on a weighted sample of 5,833 young women aged 15–24 years who reported their age at first sexual intercourse. To focus on recent experiences among young women and maintain age specificity, women aged 25–49 years were excluded from the analysis.

### Study variables and measures

#### Outcome variable.

The outcome variable for this study was the timing of first sexual experience among young women. The time-to-event variable was defined as the age (in years) at first sexual experience, measured from birth to the reported age at first sex. For respondents who had not experienced sexual intercourse at the time of the survey, their observations were treated as right-censored, with the censoring time defined as their age at the time of the interview. A binary event indicator variable was constructed to distinguish between respondents who had experienced sexual intercourse and those who had not and were therefore right-censored. Respondents who reported having had sexual intercourse were coded as 1 (event occurred), while those who had not initiated sexual activity by the time of the survey were coded as 0 (censored). This specification allowed for the application of the Cox proportional hazards regression model to assess factors associated with the timing of first sexual experience.

#### Independent variables.

Sixteen independent variables were considered in this study. These variables were selected based on the review of literature which showed their theoretical significance in determining the timing of sexual experience among young women [10,31–34]. The independent variables included age, education level, residence, province, sex of the head of household, employment status, wealth status, current marital status, religion, listening to radio, access to media, reading newspaper, watching television, literacy, tobacco use, and ownership of a mobile phone. Current age group was included as a covariate to account for differences in exposure time, as older respondents have had a longer duration at risk of sexual experience than the younger respondents [35]. The detail description of the variables is presented in Table 1.

**Table 1:** Definition of individual and household level predictor variables.

No.	Variable	Measurement
1	Age	Age of young women at the time of survey; ranged from 1 = 15–19 years; 2 = 20–24 years.
2	Education Level	Highest level of education attained: 0 = No education; 1 = Primary; 2 = Secondary, 3 = Higher.
3	Residence	Place of residence: 1 = Urban; 2 = Rural.
4	Province	Region/province of residence: 1 = Central; 2 = Copperbelt; 3 = Eastern; 4 = Luapula; 5 = Lusaka; 6 = Muchinga; 7 = Northern; 8 = North Western; 9 = Southern; 10 = Western.
5	Sex of head of household	Sex of head of household where young women lives: 1 = Male; 2 = Female
6	Employment Status	Whether respondent is currently working: 0 = No; 1 = Yes.
7	Wealth Status	Household economic status: 1 = Poor; 2 = Middle; 3 = Rich.
8	Current Marital Status	Marital status at the time of the survey: 0 = Not married; 1 = Married.
9	Religion	Religious affiliation: 1 = Catholic; 2 = Protestant; 3 = Other.
10	Listening to Radio	Listens to radio: 0 = No; 1 = Yes
11	Access to Media	Access to any form of media (radio, TV, newspaper): 0 = No; 1 = Yes.
12	Reading Newspaper	Reads newspaper or magazine: 0 = No; 1 = Yes.
13	Watching Television	Watches television: 0 = No; 1 = Yes. From v384b.
14	Literacy	Ability to read a full or part sentence: 0 = Cannot read; 1 = Can read.
15	Tobacco Use	Use of tobacco: 0 = Non-smoker; 1 = Smoker.
16	Ownership of Mobile Phone	Whether respondent owns a mobile phone: 0 = No; 1 = Yes.

### Statistical analysis

Stata was used for all analyses. Frequency and percentage distributions were used to describe the characteristics of young women in the sample. The frequency distributions were weighted to account for the complex survey design. The Kaplan–Meier curve was used to estimate the survival probability of first sexual experience by age 24 among young women in the sample. The log-rank test was used to determine statistically significant differences between groups for all independent variables. Cox proportional hazards regression was used because it effectively handles censored data from young women who had not yet experienced their first sexual intercourse by the time of the survey (right-censored). Age at first sexual experience was treated as the time-to-event variable. Unadjusted hazard ratios (UHRs), adjusted hazard ratios (AHRs), and their corresponding 95% confidence intervals (CIs) were calculated to assess the strength and direction of associations.

Survey weights were applied using the “svy” command to account for the DHS’s complex sampling design. This approach adjusted for clustering and unequal probabilities of selection, ensuring nationally representative and unbiased estimates. Kaplan–Meier estimation and Cox proportional hazards regression were used because they are appropriate for analyzing time-to-event data and can properly handle right-censored observations common in DHS data. Survival analysis is suitable because some respondents had not yet initiated sexual activity at the time of the survey; these individuals were retained in the analysis as right-censored cases, with age at interview serving as the censoring time. This approach allows for more accurate estimation of the timing of sexual experience while making full use of the available data [36,37].

A semi-parametric model frequently used in medical research to examine the relationship between respondents’ survival time and one or more predictor factors is the Cox proportional hazard model, or Cox model. We can investigate how specific characteristics affect the likelihood that a given event will occur at a given time using the Cox model. The predictor variables are covariates, and the rate is also referred to as the hazard rate. The following is the Cox model:


$$\begin{array}{c} h(t)=h_{\text{0}\;}(t)\times exp(b_{\text{1}}x_{\text{1}}+b_{\text{2}}x_{\text{2}}+\ldots +b_{p}x_{p} \end{array}$$

where;

t is the survival time;

h(t) is the hazard function;

$b_{p}$ are the coefficients that measures the impact of the p -covariates;

$h_{\text{0}\;}(t)$ is the baseline hazard. It is the value of the hazard if all the x are equal to zero.

Hazard ratios (HR) are the numbers () exp i b. When the covariate value rises, the event hazard rises and, consequently, the survival time falls, as indicated by an HR greater than one. In conclusion: HR = 1 indicates no effect, HR < 1 indicates a decrease in the hazard, and HR > 1 indicates an increase in the danger. A covariate is considered a positive prognosis factor if its HR is less than 1, and a bad prognostic factor if its HR is larger than 1. A reference level typically the first or last level of the variable is selected for categorical explanatory variables, and the other levels of the explanatory variable are compared to the reference level. The Cox model assumes that the hazard ratio of any two individuals is constant over time.

The proportional hazards assumption for the Cox proportional hazards regression model was assessed using log–log survival plots. The plots showed some moderate deviations from parallelism across certain covariate categories, indicating a potential mild violation of the proportional hazards assumption. However, the observed differences between the survival curves were relatively small and were not considered substantial enough to compromise the validity of the model. Previous methodological evidence by Austin and Giardiello (2025) suggests that moderate violations of the proportional hazards assumption are unlikely to meaningfully affect model estimates or interpretation. Therefore, the Cox proportional hazards model was considered appropriate for the analysis [38].

## Results

### Characteristics of the study participants

Table 2 presents the sociodemographic characteristics of young women included in the study. A total of 5,833 young women aged 15–24 years were included in the analysis. More than half (56%) of the respondents were aged 15–19 years. About 57% had attained secondary-level education, while only 3% had attained higher education. The majority of the respondents resided in rural areas (51%). Most young women (68%) came from male-headed households, and only 28% were employed. Almost half (45%) of the respondents were from rich households, while more than two-thirds (67%) reported never having been married. Regarding religion, the largest proportion identified as Protestant (85%). Among respondents with access to media, 15% listened to the radio, 6% read newspapers, 15% watched television, and 19% reported general access to media. Most respondents were literate (76%), nearly all (99%) were non-smokers, and 43% owned a mobile phone.

**Table 2:** Sociodemographic distribution of the young women (2024 ZDHS).

Variable	Weighted Frequency N = 5833	Weighted Percentage (%)	Median Survival Time (inter-Quartile Range - IQR)	Log Rank p-Value
**Age Group**				0.026
15-19	3,292	56.4	17 (15, 19)	
20-24	2,540	43.6	17 (15, 19)	
**Education Status**				0.000
No Education	201	3.5	15 (15, 17)	
Primary	2,124	36.4	16 (15, 18)	
Secondary	3,333	57.2	17 (16, 19)	
Higher	173	3.0	19 (17, 21)	
**Residence**				0.000
Urban	2,863	49.1	18 (16, 20)	
Rural	2,969	50.9	16 (15, 18)	
**Province**				0.000
Central	683	11.7	16 (15, 18)	
Copperbelt	833	14.3	18 (16, 21)	
Eastern	711	12.2	16 (15, 18)	
Luapula	457	7.8	17 (16, 19)	
Lusaka	976	16.7	18 (16, 20)	
Muchinga	274	4.7	17 (16, 18)	
Northern	458	7.9	17 (16, 19)	
North Western	391	6.7	16 (15, 18)	
Southern	692	11.9	16 (15, 18)	
Western	358	6.1	15 (15, 17)	
**Sex of Household Head**				0.044
Male	3,966	68.0	17 (15, 18)	
Female	1,866	32.0	17 (15, 18)	
**Employment Status**				0.003
Not working	4,170	71.5	17 (15, 19)	
Working	1,663	28.5	17 (15, 18)	
**Wealth Index**				0.000
Poor	2,079	35.6	16 (15, 17)	
Middle	1,151	19.7	16 (15, 18)	
Rich	2,603	44.6	18 (16, 20)	
**Marital Status**				0.000
Never Married	3,891	66.7	18 (16, 20)	
Ever Married	1,941	33.3	16 (15, 17)	
**Religion**				0.012
Catholics	823	14.1	17 (15, 19)	
Protestant	4,982	85.4	17 (15, 19)	
Other	27	0.5	15 (14, 17)	
**Listening to Radio**				0.000
No	4,715	85.5	17 (15, 18)	
Yes	1,117	14.5	17 (16, 19)	
**Access to Media**				0.000
No	4,179	80.8	17 (15, 18)	
Yes	1,654	19.2	17 (16, 19)	
**Reading Newspaper**				0.000
No	5,473	93.8	17 (15, 19)	
Yes	360	6.2	18 (16, 20)	
**Watching TV**				0.000
No	4,946	84.8	17 (15, 18)	
Yes	887	15.2	18 (16, 20)	
**Literacy**				0.000
Not literate	1,384	23.7	16 (15, 17)	
Literate	4,447	76.3	17 (15, 19)	
**Tobacco Use**				0.152
None smoker	5,797	99.4	17 (15, 19)	
Smoker	36	0.6	16 (14, 18)	
**Own a phone**				0.000
No	3,120	53.5	17 (15, 18)	
Yes	2,712	46.5	17 (15, 19)	

### The time to first sexual experience among young women in Zambia

**Fig 1:**
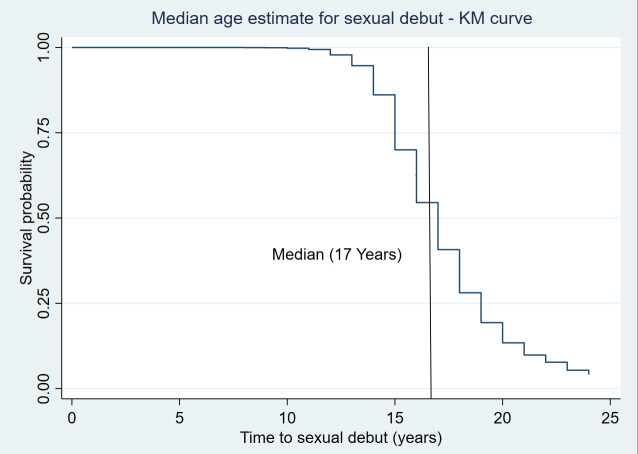
Time to first sexual experience among young women in Zambia.

Fig 1 showed that 4109 (70% [95% CI: 0.71 – 0.73]) of the young women had their first sexual experience before the age of 25. The overal median age to first sexual experience was 17 years.

### The time to first sexual experience among young women in Zambia by age, education level, employment and education

**Fig 2:**
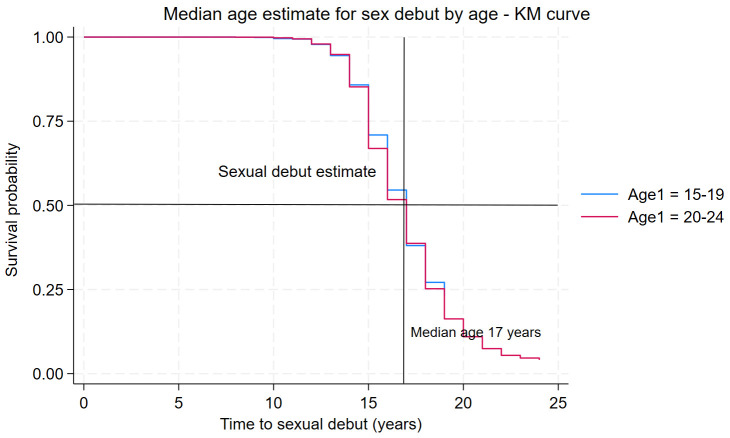
Time to first sexual experience among young women by age.

**Fig 3:**
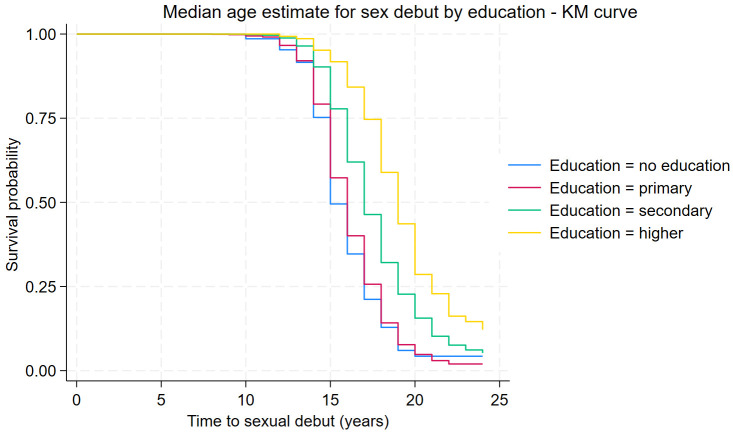
Time to first sexual experience among young women by education status.

**Fig 4:**
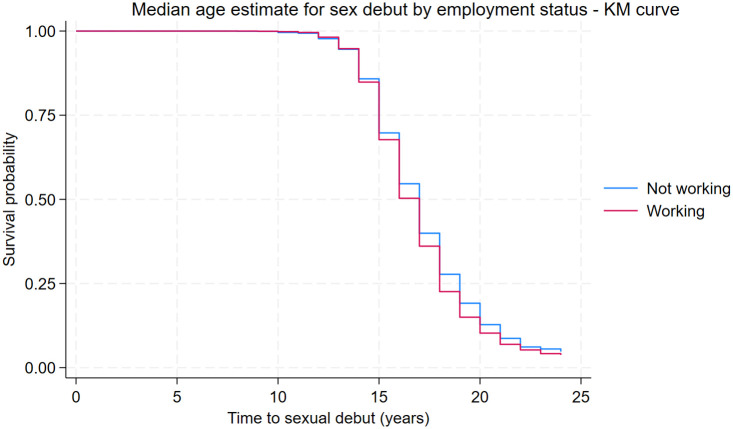
Time to first sexual experience among young women by employment status.

**Fig 5:**
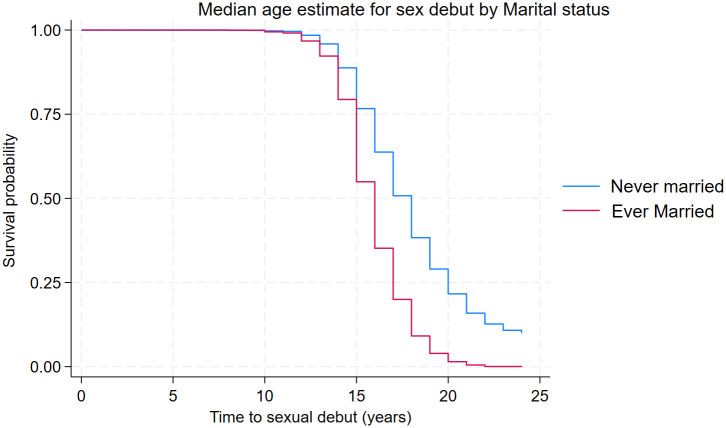
Time to first sexual experience among young women by marital status.

Different demographic characteristics were used to compare survival time using the Log-rank test and Kaplan-Meier (KM) survival curves. The results show that women aged 20–24 years have a higher probability of experiencing the event (first sexual experience) than those aged 15–19 years (Fig 2). Young women with no education are more likely to initiate sexual activity earlier than those with secondary and higher education (Fig 3). Young women who are working have a higher probability of experiencing the event compared to those who are not working (Fig 4). Similarly, ever-married young women have a higher probability of first sexual experience occurring earlier than their never-married counterparts (Fig 5).

### Bivariable cox regression analysis for determinants of time to first sexual experience among young women in Zambia

Table 3 presents the results of the bivariable Cox regression analysis on the determinants of first sexual experience among women in Zambia. The findings indicate that age, education status, residence, province, sex of household head, employment status, wealth index, marital status, religion, listening to the radio, access to media, reading newspapers, watching television, literacy, and phone ownership were significantly associated with sexual experience at *p* < 0.05.

**Table 3:** Bivariable cox regression analysis for determinants of time to first sexual experience among young women (2024 ZDHS).

Variable	Crude Harzard Ratio (CHR)	P-Value	Confidence Interval (95% CI)Lower - Upper
**Age Group**			
15-19	1		
20-24	1.08	0.026	1.01 - 1.15
**Education Status**			
No Education	2.99	0.000	2.36 - 3.78
Primary	2.7	0.000	2.23 - 3.26
Secondary	1.57	0.000	1.31 - 1.90
Higher	1		
**Residence**			
Urban	1		
Rural	1.83	0.000	1.72- 1.95
**Province**			
Central	1.46	0.000	1.29 - 1.67
Copperbelt	0.83	0.009	0.72 - 0.95
Eastern	1.74	0.000	1.53 - 1.98
Luapula	1.24	0.003	1.08 - 1.43
Lusaka	1		
Muchinga	1.33	0.000	1.15 - 1.53
Northern	1.16	0.037	1.01 - 1.33
North Western	1.79	0.000	1.55 - 2.05
Southern	1.92	0.000	1.69 - 2.18
Western	2.07	0.000	1.79 - 2.37
**Sex of Household Head**			
Male	1		
Female	0.94	0.045	0.87 - 1.00
**Employment Status**			
Not working	1		
Working	1.1	0.003	1.03 - 1.17
**Wealth Index**			
Poor	1		
Middle	0.81	0.000	0.75 - 0.87
Rich	0.45	0.000	0.42 -0.49
**Marital Status**			
Never Married	1		
Ever Married	2.15	0.000	2.02 - 2.28
**Religion**			
Catholics	1		
Protestant	1.12	0.009	1.03 - 1.23
Other	1.50	0.069	0.97 - 2.32
**Listening to Radio**			
No	1		
Yes	0.86	0.000	0.81 - 0.94
**Access to Media**			
No	1		
Yes	0.75	0.000	0.70 - 0.81
**Reading Newspaper**			
No	1		
Yes	0.71	0.000	0.62 - 0.81
**Watching TV**			
No	1		
Yes	0.67	0.000	0.61 - 0.73
**Literacy**			
Not literate	1		
Literate	0.57	0.000	0.53 - 0.61
**Tobacco Use**			
None smoker	1		
Smoker	1.29	0.136	0.92 - 1.81
**Own a phone**			
No	1		
Yes	0.84	0.000	0.79 - 0.90

### Multivariable cox regression analysis for determinants of time to first sexual experience among young women in Zambia

Table 4 presents the results of the multivariable Cox regression analysis of determinants of time to first sexual experience among young women. From the Cox regression model, women aged 20–24 years had a lower hazard of early sexual experience (AHR = 0.75, 95% CI: 0.70–0.80) than those aged 15–19 years. Women with no education had a 45% higher hazard of early sexual experience (AHR = 1.45, 95% CI: 1.15–1.84), while those with primary education had a 44% higher hazard (AHR = 1.44, 95% CI: 1.22–1.72), and those with secondary education had a 19% higher hazard (AHR = 1.19, 95% CI: 1.02–1.40), compared to those with higher education. Women living in rural areas were more likely to experience early sexual debut than those in urban areas. Women from female-headed households had a 14% higher hazard of early sexual experience (AHR = 1.14, 95% CI: 1.06–1.23) compared to those from male-headed households. Employed women were also 13% more likely to experience early sexual debut (AHR = 1.13, 95% CI: 1.05–1.21) compared to unemployed women. Women from rich households were less likely to experience early sexual debut (AHR = 0.75, 95% CI: 0.67–0.85) compared to those from poor households. Married women had a higher hazard of early sexual experience (AHR = 2.03, 95% CI: 1.88–2.18) than those who had never married. Literacy was associated with a lower hazard (AHR = 0.87, 95% CI: 0.80–0.94). Finally, phone ownership was associated with a slightly higher hazard of early sexual experience (AHR = 1.16, 95% CI: 1.00–1.17).

**Table 4:** Multivariable cox regression analysis for determinants of time to first sexual experience among young women (2024 ZDHS).

Variable	Adjusted Harzard Ratio (AHR)	P-Value	Confidence Interval (95% CI)Lower - Upper
**Age Group**			
15-19	1		
20-24	0.75	0.000	0.70 - 0.80
**Education Status**			
No Education	1.45	0.002	1.15 - 1.84
Primary	1.44	0.000	1.22 - 1.72
Secondary	1.19	0.027	1.02 - 1.40
Higher	1		
**Residence**			
Urban	1		
Rural	1.18	0.001	1.07 - 1.30
**Province**			
Central	1.03	0.742	0.88 - 1.19
Copperbelt	0.78	0.001	0.68 - 0.91
Eastern	1.03	0.711	0.88 - 1.20
Luapula	0.81	0.017	0.68 - 0.96
Lusaka	1		
Muchinga	0.89	0.088	0.77 - 1.02
Northern	0.77	0.001	0.66 - 0.90
North Western	1.37	0.000	1.17 - 1.60
Southern	1.5	0.000	1.30 - 1.74
Western	1.55	0.000	1.33 - 1.81
**Sex of Household Head**			
Male	1		
Female	1.14	0.000	1.06 - 1.23
**Employment Status**			
Not working	1		
Working	1.13	0.001	1.05 - 1.21
**Wealth Index**			
Poor	1		
Middle	0.98	0.658	0.89 - 1.07
Rich	0.75	0.000	0.67 - 0.85
**Marital Status**			
Never Married	1		
Ever Married	2.03	0.000	1.88 - 2.18
**Religion**			
Catholics	1		
Protestant	1.05	0.253	0.96 - 1.14
Other	1.58	0.007	1.13 - 2.20
**Listening to Radio**			
No	1		
Yes	1.04	0.365	0.96 - 1.12
**Access to Media**			
No	–	–	–
Yes	–	–	–
**Reading Newspaper**			
No	1		
Yes	0.94	0.322	0.83 - 1.06
**Watching TV**			
No	1		
Yes	0.93	0.125	0.84 - 1.02
**Literacy**			
Not literate	1		
Literate	0.87	0.001	0.80 - 0.94
**Tobacco Use**			
None smoker	1		
Smoker	1.03	0.882	0.73 - 1.44
**Own a phone**			
No	1		
Yes	1.16	0.000	1.09 - 1.24

Note: Access to media was not included in the model due to multi-collinearity.

## Discussion

This study aimed to identify the factors associated with the time to first sexual experience among young women in Zambia aged 15–24 years. The findings revealed that 70% of the young women had their first sexual experience before their 25th birthday. The probable reason could be the prevailing social and cultural norms in Zambia where sexual initiation commonly occurs during late adolescence and early adulthood, leading most young women to experience sexual intercourse before age 25 [2,19]. Comparable studies conducted in Mozambique found that 80% of young women had their first sexual encounter before age 25 [39], while a study from Eswatini reported a prevalence of 70% [40]. The discrepancies in sexual experiences among young women may be due to various factors such as social and religious norms which make emphasis on the importance of abstinence until marriage especially for young women which may have contributed to later sexual experiences. There is an urgent need for government to strengthen school-based comprehensive sexuality education to delay sexual initiation and improve the socio-economic well-being of young women [10,31,32,41].

The study also examined the determinants of time to first sexual experience and the results showed that demographic, socioeconomic, and informational factors significantly influence the timing of sexual experience. These factors included age, education, province, household characteristics, and exposure to information. The study further found that young women aged 20–24 years had reduced hazard ratio for early sexual experience than those aged 15–19 years. The possible reason for this result could be because younger adolescents (15–19 years) are more likely to initiate sex earlier due to peer influence, curiosity, and limited decision-making autonomy, whereas older young women (20–24 years) may delay sexual experience due to greater maturity and life priorities such as education or work [40,42]. This finding is similar with results from Mozambique and Sierra Leone, where older age was associated with a lower risk of initiating sexual activity [15,39].

Regarding education, young women with no education, and those with primary or secondary education, had an increased hazard ratio for sexual initiation compared to those with higher education. The plausible explanation for this finding is that higher levels of education among young women may enhance their autonomy and decision-making capacity, thereby enabling them to make more informed choices and ultimately delaying the timing of first sexual experience [39,43]. Studies conducted in SSA found similar result that higher education was associated with lower chances for first sexual experience [33,34,43].

In terms of employment, young women who were employed had an increased hazard ratio for early sexual experience compared to those who were unemployed. A possible explanation is that employment exposes young women to broader social networks and environments where sexual activity may be more likely to occur [5,34]. This contrasts with a study conducted in Eswatini, which found that employed women had a lower risk of early sexual activity [40]. With respect to wealth status, young women from rich households had a reduced hazard ratio for early sexual experience compared to those from poor households. One possible reason for the observed result could be that women from wealthier households are less likely to initiate early sexual activity due to better access to education and information. They are more likely to stay in school longer, which delays sexual experience. Greater economic security also reduces engagement in transactional sex. Additionally, higher parental supervision and future aspirations contribute to more cautious behavior [34]. This is consistent with a study conducted in Nigeria, which found that individuals from wealthier households had a lower risk of early sexual experience [16]. Additionial, marital status was also an important determinant. Ever-married women had an increased hazard ratio for early sexual experience compared to those who were never married.

Regarding marital status, ever-married women are more likely to have already initiated sexual activity due to the nature of marital unions, which often formalize or legitimize sexual relationships. This increases their likelihood of reporting earlier sexual experience compared to never-married women. Additionally, social and cultural norms surrounding marriage in some settings may encourage early union formation, which in turn is associated with earlier sexual initiation [39]. This finding is in line with evidence from Sierra Leone, which similarly showed that married women were at a higher risk of early sexual experience [15] Literate young women were found to have a lower hazard of early sexual initiation compared to their non-literate counterparts. A plausible explanation for this finding is that literacy enhances access to, and comprehension of, health-related information, including sexual and reproductive health messages. The improved ability to read and interpret information may enable literate young women to better understand the potential consequences of early sexual activity, such as unintended pregnancy and sexually transmitted infections, thereby encouraging more cautious decision-making [44,45]. The result from this study contradicts with the study conducted in Kenya which established to find no relationship between first sexual experience and literacy [42].

Religious affiliation was found to be associated with the timing of sexual experience, where young women belonging to other religious groups had a higher likelihood of early sexual experience than the Catholic counterparts. A plausible explanation for this finding may lie in differences in religious teachings, doctrines, and the extent to which behavioural norms around sexuality are emphasized and enforced across religious denominations. Some religious traditions may place stronger emphasis on abstinence, sexual restraint, and moral teachings regarding premarital sexual activity, which may contribute to delayed sexual initiation among adherents [46,47]. A study conducted in Ethiopia discovered that there was no significant relationship between religion and first sexual experience [48]. Phone ownership was associated with an increased hazard of earlier sexual initiation among young women. A plausible explanation for this finding is that mobile phone access may increase exposure to a wider range of unregulated digital content, including sexually explicit materials, which may in turn influence attitudes, perceptions, and curiosity regarding sexual activity. Increased connectivity through mobile phones may also facilitate peer communication and social media interactions that can normalize or accelerate discussions around sexual relationships, potentially contributing to earlier sexual experience [49,50]. The result from this study was consistent with study conducted in Uganda which showed that mobile phone ownership was associated with first sexual experience [51].

The results from this study identified several implications for adolescent sexual and reproductive health (SRH) policies and programs in Zambia. The protective effect of education on delayed sexual experience emphasizes the need to strengthen the implementation of existing education and gender policies that promote girls education. The Ministry of Education, through the Keeping Girls in School (KGS) initiative and other complementary programs, should continue addressing school dropouts caused by early pregnancies, poverty, and child marriage. Expanding access to secondary and tertiary education for girls, particularly in rural and underserved areas, is essential for empowering young women and reducing early sexual experience. Integrating Comprehensive Sexuality Education (CSE) more effectively within the school and community settings can further equip adolescents with accurate information and life skills to make informed decisions.

### Limitation of the study

Several limitations where identified in the study. The cross-sectional ZDHS data used in this study does not follow the same people over time, it naturally restricts the capacity to make inferences about causality. In this case, survival analysis was employed to estimate the time of first sexual experience while accounting for censored data, even though it is often used for longitudinal data. However, recall bias may be introduced by self-reported measurements, and the results may be impacted by unmeasured confounders.

## Conclusion

This study examined the determinants of time to first sexual experience among young women in Zambia using Kaplan–Meier survival analysis and Cox proportional hazards regression. The findings show that a substantial proportion of young women initiated sexual activity before the age of 25, with a median age at first sexual experience of 17 years. The multivariable Cox regression analysis identified several factors significantly associated with earlier sexual initiation. These included younger age, lower educational attainment, rural residence, female-headed households, being ever married, being in employment, lower wealth status, and exposure to media, particularly phone ownership. In contrast, higher levels of education and literacy were associated with delayed sexual initiation. These findings suggest variation in the timing of sexual initiation across different socioeconomic and demographic characteristics among young women in Zambia. Programmatic efforts may consider strengthening girls’ education, improving access to socioeconomic opportunities, and enhancing sexual and reproductive health information, including through appropriate use of media platforms, particularly in disadvantaged settings.
