# Supplementary material for: Time to first sexual experience and its predictors among young women (15–24 years) in Zambia: Evidence from 2024 Demographic and Health Survey
Source: PLOS Glob Public Health. 2026 Jun 22;6(6):e0006687. doi: 10.1371/journal.pgph.0006687 (PMC13286187; doi:10.1371/journal.pgph.0006687)
Supplement: Attachment — Submitted filename: Time to first sexual debut.docx [file pgph.0006687.s001.docx]

**Review comments**

**My comments on the manuscript entitled: “Time to first sexual debut and its predictors among young women (15–24 years) in Zambia: Evidence from 2024 Zambia Demographic and Health Survey**.”

This manuscript examines the timing of first sexual debut among young women aged 15–24 in Zambia using data from the 2024 Zambia Demographic and Health Survey (ZDHS). The authors employ Kaplan–Meier survival analysis, log-rank tests, and Cox proportional hazards regression to identify determinants of sexual debut. The topic is relevant to sexual and reproductive health (SRH), adolescent health policy, and HIV/STI prevention in sub-Saharan Africa. Overall, the manuscript has potential but requires improvement in certain areas.

1. The manuscript does not present a conceptual or theoretical framework guiding the analysis of sexual debut. Sexual debut is widely studied using frameworks such as Social Ecological Model, Health Belief Model, Life Course Perspective, etc. Without a guiding framework, the selection of variables appears largely descriptive. The authors should introduce a conceptual model explaining how socioeconomic, demographic and informational factors influence sexual initiation. This will also strengthen the Introduction as it is current somewhat brief.

2. Although the introduction provides statistics on STIs and teenage pregnancy, it does not clearly articulate what specific knowledge gap this study fills, why Zambia specifically requires further investigation and how this study advances existing research.

3. again, even though the authors applied Kaplan–Meier and Cox regression, the justification for survival analysis is not adequately explained. Why is survival analysis appropriate for DHS data? Was age at interview used as censoring time?

4. some interpretations in the results section appears problematic. For example, the authors state that “Females aged 20–24 had reduced hazards of sexual debut compared to those aged 15–19. However, this interpretation may reflect age cohort effects rather than causal relationships as the authors assumes. Similarly, the interpretation of marital status as a predictor of sexual debut is conceptually questionable because sexual debut often precedes marriage. The authors should reconsider how these variables are interpreted.

5. The discussion is largely descriptive and simply repeats results with limited analytical depth. A stronger discussion should explain why these associations exist and link findings to existing literature.

6. finally, the authors should read through for grammar and typographical errors. references 10 and 27 appear duplicated. The phrase “young females” could be replaced with “young women” for consistency with global health literature.
