# Supplementary material for: Time to first sexual experience and its predictors among young women (15–24 years) in Zambia: Evidence from 2024 Demographic and Health Survey
Source: PLOS Glob Public Health. 2026 Jun 22;6(6):e0006687. doi: 10.1371/journal.pgph.0006687 (PMC13286187; doi:10.1371/journal.pgph.0006687)
Supplement: Attachment — Submitted filename: peer_review_report_sexual_debut_zambia.docx [file pgph.0006687.s002.docx]

**Peer Review Report**

This manuscript addresses an important public health issue by examining the timing of first sexual debut among young women in Zambia using nationally representative data from the 2024 Zambia Demographic and Health Survey. The study is timely and has strong potential to contribute to understanding factors that influence early sexual initiation and to inform sexual and reproductive health policies and interventions in Zambia. The use of survival analysis techniques such as Kaplan–Meier curves and Cox proportional hazards models is appropriate for analysing the timing of sexual debut. The authors are commended for undertaking a study with important policy implications. However, several areas of the manuscript require clarification, methodological refinement, and language editing in order to strengthen the scientific rigor and clarity of the paper. The comments below are provided to support improvement of the manuscript.

Title: The title is relevant and communicates the topic clearly; however, it could be improved for clarity and precision. The phrase “time to first sexual debut” is somewhat repetitive because the term debut already implies the first occurrence. A clearer formulation would be “age at sexual debut” or “time to first sexual intercourse.”

Abstract – Introduction: The sentence stating that “little is known about this area of research in Zambia” is vague. It would be more appropriate to clarify that limited recent evidence exists, particularly using nationally representative data from the 2024 Zambia Demographic and Health Survey.

Abstract – Methods: The phrase “with the level of significance of 0.05” is verbose and should be replaced with the standard statistical phrasing “Statistical significance was set at p < 0.05.”

Abstract – Conclusion: The conclusion is relevant but slightly overreaches beyond the results presented in the abstract. It would be preferable for the conclusion to align closely with the predictors actually reported in the abstract.

Abstract – Keywords and formatting: The term “Key word” should be corrected to “Keywords,” and capitalization inconsistencies throughout the abstract should be addressed.

Introduction – Grammar: The statement “the median age for sexual debut was 16 year” should be corrected to “16 years.”

Introduction – Variable capitalization: Variables such as Age, Education, religion, and place of residence should be consistently written in lowercase when used as general descriptors.

Introduction – Terminology: The manuscript alternates between the terms “sexual debut,” “sexual experience,” and “first sexual intercourse.” A single consistent term should be used throughout the manuscript.

Methods – Outcome variable: The description of the outcome variable requires clarification. While the authors indicate that age at first sexual intercourse was used as the time-to-event variable, they also mention constructing a binary variable for sexual experience. Cox proportional hazards models require both a time variable and an event indicator. The authors should clearly specify the time variable and censoring indicator.

Methods – Censoring: The handling of censored observations should be explained more clearly. For respondents who had not yet experienced sexual debut, the censoring time should correspond to the respondent’s age at interview.

Methods – Age variable: The inclusion of current age group (15–19 vs 20–24) as an explanatory variable requires justification because age at first sexual intercourse is already used as the time variable in the survival model.

Results – Education interpretation: The Kaplan–Meier interpretation of education appears inconsistent with the results. The tables indicate that higher education is associated with delayed sexual debut, whereas the narrative suggests the opposite.

Results – Marital status: The interpretation of marital status requires caution because sexual debut often occurs prior to marriage. Current marital status may therefore reflect reverse temporality.

Reporting p-values as “<0.05” limits the information available to readers. Providing the exact p-value (e.g., *p = 0.032*) improves transparency and allows readers to better assess the strength of statistical evidence. In addition, the p-value should correspond to the overall comparison across categories and should therefore be presented clearly in the p-value column associated with the variable.

Results – Hazard ratio interpretation: The narrative should refer to the hazard of earlier sexual debut rather than simply “hazard of sexual debut.”

Results – Terminology consistency: The manuscript alternates between “young females,” “young women,” and “females.” It would be preferable to consistently use the term “young women.”

Results – p-value formatting: The log-rank p-value for phone ownership is reported as <0.000, which should instead be written as <0.001.

Results – Small subgroup caution: Estimates for the “Other” religion category should be interpreted cautiously due to the very small sample size.

Statistical model assumptions: The Cox proportional hazards model assumes proportional hazards, meaning hazard ratios remain constant over time. While the manuscript explains this assumption theoretically, it does not report whether it was tested. The authors should indicate whether diagnostics such as Schoenfeld residual tests or log–log survival plots were conducted.

Discussion – Study population: The opening sentence incorrectly states that the study included women aged 20–24 years. The analysis actually includes women aged 15–24 years.

Discussion – Age interpretation: The discussion should reconsider interpreting age group as a determinant of sexual debut given the time-scale structure of the survival model.

Conclusion: The conclusion provides a useful summary but should ensure that only predictors significant in the multivariable model are emphasized and causal language is avoided.

References: Reference 27 appears to duplicate Reference 10 and should be removed. The remaining references should be renumbered accordingly.
