# Supplementary material for: Time to first sexual experience and its predictors among young women (15–24 years) in Zambia: Evidence from 2024 Demographic and Health Survey
Source: PLOS Glob Public Health. 2026 Jun 22;6(6):e0006687. doi: 10.1371/journal.pgph.0006687 (PMC13286187; doi:10.1371/journal.pgph.0006687)
Supplement: Attachment — Submitted filename: Rebuttal letter_final.docx [file pgph.0006687.s003.docx]

The Chief Editor,

Plos One Global Public Health

11^th^ May 2026

**Sub: Response to Decision Letter: PGPH-D-26-00559: Time to first sexual debut and its predictors among young women (15-24 years) in Zambia: evidence from 2024 Zambia Demographic and Health Survey**

Refer to the above subject matter.

We are grateful to the editor and reviewers for their time and constructive comments on our manuscript. We have taken into consideration the second round of rviews and suggestions the editor, and we would like to submit a revised version of the manuscript for consideration by the journal. To reflect changes or amendments made in the revised version of the manuscript, changes or amendments have been highlighted in the initial version of the manuscript. In addition, we provide a point-by-point response explaining how we have addressed the editors comments in the section following this one. We are looking forward to hearing the results of your evaluation.

Yours sincerely,

Lead author

On behalf of co-authors

**Title: Time to first sexual debut and its predictors among young women (15-24 years) in Zambia: evidence from 2024 Zambia Demographic and Health Survey**

| **Journal Requirements** | | |
| --- | --- | --- |
| **Editors Remarks** | **Authors Response** | **Reference** |
| 1. Please amend your online Financial Disclosure statement. If you did not receive any funding for this study, please simply state: "The authors received no specific funding for this work." | Financial disclose has been included in the online system | **Online system** |
| Please update your online Competing Interests statement. If you have no competing interests to declare, please state: "The authors have declared that no competing interests exist." | The competing interest statement has been included in the online system | Online system |
| Please provide separate main figure files in .tif or .eps format only and ensure that all files are under our size limit of 10MB. For more information about how to convert your figure files please see our guidelines: <https://journals.plos.org/globalpublichealth/s/figures> | All the files have been formatted .tif format | **Figure 1 to Figure 5** |
|  |  |  |
| **Reviewer 1** | | |
| **Questions and Comments** | **Authors Response** | **Reference** |
| Reviewer #1: This manuscript examines the timing of first sexual debut among young women aged 15–24 in Zambia using data from the 2024 Zambia Demographic and Health Survey (ZDHS). The authors employ Kaplan–Meier survival analysis, log-rank tests, and Cox proportional hazards regression to identify determinants of sexual debut. The topic is relevant to sexual and reproductive health (SRH), adolescent health policy, and HIV/STI prevention in sub-Saharan Africa. Overall, the manuscript has potential but requires improvement in certain areas. | **We are grateful for your favourable**  **comments and remarks. We would like to express our appreciation for your valuable expertise and the time you dedicated to our work.** |  |
| 1. The manuscript does not present a conceptual or theoretical framework guiding the analysis of sexual debut. Sexual debut is widely studied using frameworks such as Social Ecological Model, Health Belief Model, Life Course Perspective, etc. Without a guiding framework, the selection of variables appears largely descriptive. The authors should introduce a conceptual model explaining how socioeconomic, demographic and informational factors influence sexual initiation. This will also strengthen the Introduction as it is current somewhat brief. | Thank you your comment. We have included the section on theorectical model used for the study. | **Line 83 – Line 112** |
| 2. Although the introduction provides statistics on STIs and teenage pregnancy, it does not clearly articulate what specific knowledge gap this study fills, why Zambia specifically requires further investigation and how this study advances existing research. | Thank you for your comment. More clarity has been provided to strength the identified gap and how this study will fill the gap | **Line 70 – Line 80** |
| 3. Again, even though the authors applied Kaplan–Meier and Cox regression, the justification for survival analysis is not adequately explained. Why is survival analysis appropriate for DHS data? Was age at interview used as censoring time? | Thank you the comment. The justification for using survival analysis has been included in the study.  The varaible that was used for censoring was included in the ,methodology | **Line 172 – Line 177**  **Line 137 – Line 146** |
| 4. some interpretations in the results section appears problematic. For example, the authors state that “Females aged 20–24 had reduced hazards of sexual debut compared to those aged 15–19. However, this interpretation may reflect age cohort effects rather than causal relationships as the authors assumes. Similarly, the interpretation of marital status as a predictor of sexual debut is conceptually questionable because sexual debut often precedes marriage. The authors should reconsider how these variables are interpreted. | Thank you for the comment. The intepretation of age and mrital status variables have been correctly interpreted. | **Line 264 – Line 265**  **Line 274 - Line 276** |
| 5. The discussion is largely descriptive and simply repeats results with limited analytical depth. A stronger discussion should explain why these associations exist and link findings to existing literature. | Thank you for your observations and Comment.  - The analytical depth has been provided in the discusion section  - We have made improvements in the discussion of results by ensuring that all findings are linked to existing literature | **Whhole discussion section** |
| 6. finally, the authors should read through for grammar and typographical errors. references 10 and 27 appear duplicated. The phrase “young females” could be replaced with “young women” for consistency with global health literature. | Thank you for the observation, the two reference have been corrected and the manuscript was subjected to professional editing. | **Whole reference section** |
| **Reviewer 2** | | |
| Reviewer #2: The authors have carried out a research on an issue of public health significance and have used the appropriate data analysis technique. There are however some concerns noted which have been highlighted below: | We are grateful for your favourable  comments and remarks. We would like to  express our appreciation for your valuable  expertise and the time you dedicated to our  work. |  |
| Comment 1. Line 23-27: “The results of the multivariable Cox regression analysis for determinants of time to first sexual experience among young females. Established that Determinants with reduced hazards ratio of timing to first sexual debut included females aged 20–24 years (AHR = 0.75, 95% CI: 0.70–0.80), Females from rich households (AHR26 = 0.75, 95% CI: 0.67–0.85), literate women (AHR = 0.87, 95% CI: 0.80–0.94)” These two sentences should be merged and shortened | Thank you for the observation. The sentence has been merged and shortened | **Line 25 – Line 27** |
| Comment 2. Line 38. “Sexual debut timing among young women continues to attract significant interest, as it marks the onset of exposure to various sexual and reproductive health outcomes” This is grammatically wrong. Instead state that “it marks the onset of exposure to adverse sexual and reproductive health outcomes”. | The suggestion has been effected | **Line 37 –Lime 38** |
| Comment 3. Line 52. “Age, Education, religion, place of residence, region” Education should start with a small letter, not a capital letter | The edit has been effected. | **Line 52** |
| Comment 4. Line 57. “When factors are identified and comprehended, this will influence policymakers and healthcare provide to develop strategies” Healthcare providers not provide | The edit has been effected. | **Line 58** |
| Comment 5. Include that you applied survey weights in the methods section | Thank you for your comment. I have included the senetences on the apsect that weights were used in this study. | **Line 170 – Line 172** |
| Comment 6. Line 176. “Females with higher education are more likely to initiate sexual activity earlier than those with no education (Figure 3)” According to the Kaplan Meiyer curve, the reverse is the case. You have not interpreted the Kaplan Meiyer curve correctly | Thank you for the observation. The correct interpretation for education variable has been provided. | **Figure 3** |
| Comment 7. “Females who are not working have a higher probability of experiencing the event compared those who are employed (Figure 4). According to the Kaplan Meiyer curve, the reverse is the case. You have not interpreted the Kaplan Meiyer curve correctly | Thank you for the observation. The correct interpretation for emploment variable has been provided. | **Figure 4** |
| Comment 8. Line 178. Similarly, never-married females have a higher probability of first sexual experience occurring earlier than their married counterparts (Figure 5). According to the Kaplan Meiyer curve, the reverse is the case. You have not interpreted the Kaplan Meiyer curve correctly. | Thank you for the observation. The correct interpretation for marital status variable has been provided. | **Figure 5** |
| Comment 9. Line 196. There appears to be an inconsistency between the Kaplan–Meier survival curves and the bivariate Cox regression results for marital status. Since both analyses are unadjusted comparisons, they would generally be expected to show a similar direction of association. The authors are encouraged to verify the coding of the event variable, the reference category used in the Cox model, and the specification of the survival time variable. Clarification of this discrepancy would strengthen the validity of the analysis. This should be done for all the variables in the bivariate analysis. | Thank you for the observation and comment. We have verifified the marital status variable and we have observed the similar direction for kaplan meier curve and bivariate indicating that ever married had a higher chance to experience the event. For survival curve the red curve is closer to the x – axis, this signifies that married young women had higher hazards of first sexual experience. |  |
| Comment 10. Multivariable cox regression analysis is however in line with the Kaplan Meiyer curves | Thank you for this observation |  |
| Comment 11. Line 220 “This study aimed to identify the factors associated with the time to first sexual experience among young females in Zambia aged 20–24 years.”  It should be 15 to 24 years. | The edit has been effected | **Line 286** |
| Comment 12. “Therefore, there is urgent need for government to take keen interest in ensuring that schools starts to offer effective sexual programs which include comprehensive sexuality education program. Which can actually promote the delay in sexual initiations and enhancing the economic and social condition for young women” These sentences should be merged but should not be too long | Thank you for your comment. The sentence has been merged and shorted | **Line 294 – Line 296** |
| Comment 13. Discussion: The discussion is very brief. Please give likely explanations for your findings e.g. on marital status, education, head of household. Other variables found significant but not discussed should be discussed e.g. use of mobile phones, literacy level. | Thank you for your observation and comment. The discussion section has been improved by providing likely exaplnnation and also discussing the results which were not included. | **Line 341 – Line 356** |
| Comment 14. Line 278. “Younger age, lower educational attainment, being from male-headed households, employment, lower wealth status, and being ever married were associated with a higher likelihood of early sexual initiation” | Even though it was not clear what exactly we were suppose to do on this comment, we have improved the phrasing of the sentence. | **Line 378 – Line 380** |
| Comment 15. The conclusion states that being from male headed households was associated with higher likelihood of early sexual initiation. However, the multivariate Cox model states the reverse.  Please go through all your tables, graphs, results section and discussion and ensure they are in line. Re-do any analysis that was not properly done, probably in the bivariate analysis.  Note that your discussion on determinants should be based on findings from the multivariate Cox proportional hazards model and not from the bivariate analysis. | Thank you for the observation. The statement with regards to sex of the household has been corrected. We have run through the analysis and checked the results section to verify the results. | **Line 379** |
| **Reviewer 3** | | |
| Reviewer #3: This manuscript addresses an important public health issue by examining the timing of first sexual debut among young women in Zambia using nationally representative data from the 2024 Zambia Demographic and Health Survey. The study is timely and has strong potential to contribute to understanding factors that influence early sexual initiation and to inform sexual and reproductive health policies and interventions in Zambia. The use of survival analysis techniques such as Kaplan–Meier curves and Cox proportional hazards models is appropriate for analysing the timing of sexual debut. The authors are commended for undertaking a study with important policy implications. However, several areas of the manuscript require clarification, methodological refinement, and language editing in order to strengthen the scientific rigor and clarity of the paper. The comments below are provided to support improvement of the manuscript. | We are grateful for your favourable  comments and remarks. We would like to  express our appreciation for your valuable  expertise and the time you dedicated to our  work. |  |
| 1. Title: The title is relevant and communicates the topic clearly; however, it could be improved for clarity and precision. The phrase “time to first sexual debut” is somewhat repetitive because the term debut already implies the first occurrence. A clearer formulation would be “age at sexual debut” or “time to first sexual intercourse.” | The edit has been effected to as comment below for consistency we agreed to use “Time to first sexual experience”. |  |
| 2. Abstract – Introduction: The sentence stating that “little is known about this area of research in Zambia” is vague. It would be more appropriate to clarify that limited recent evidence exists, particularly using nationally representative data from the 2024 Zambia Demographic and Health Survey. | Thank you for your comment. Further clarification has been provided on the statement. | **Line 14 – Line 16** |
| 3. Abstract – Methods: The phrase “with the level of significance of 0.05” is verbose and should be replaced with the standard statistical phrasing “Statistical significance was set at p < 0.05.” | The suggested edit have been effected. | **Line 21 – line 22S** |
| 4. Abstract – Conclusion: The conclusion is relevant but slightly overreaches beyond the results presented in the abstract. It would be preferable for the conclusion to align closely with the predictors actually reported in the abstract. | Thank you for your comment. The conclusion has been revised in line with the guidance you have provided. | **Line 28 – Line 30** |
| 5. Abstract – Keywords and formatting: The term “Key word” should be corrected to “Keywords,” and capitalization inconsistencies throughout the abstract should be addressed. | The suggested edits have been effected | **Line 31** |
| 6. Introduction – Grammar: The statement “the median age for sexual debut was 16 year” should be corrected to “16 years.” | The edit has been effected. | **Line 50** |
| 7. Introduction – Variable capitalization: Variables such as Age, Education, religion, and place of residence should be consistently written in lowercase when used as general descriptors. | The edit has been effected | **Line 52** |
| 8. Introduction – Terminology: The manuscript alternates between the terms “sexual debut,” “sexual experience,” and “first sexual intercourse.” A single consistent term should be used throughout the manuscript. | Thank you for the guidance. We have agreed to consistently use the term “first sexual expereince” throughout the manuscript. | **The whole manuscript** |
| 9. Methods – Outcome variable: The description of the outcome variable requires clarification. While the authors indicate that age at first sexual intercourse was used as the time-to-event variable, they also mention constructing a binary variable for sexual experience. Cox proportional hazards models require both a time variable and an event indicator. The authors should clearly specify the time variable and censoring indicator. | Thank you for the comment. Further clarification has been provided in the methods section. | **Line 137 – Line 146** |
| 10. Methods – Censoring: The handling of censored observations should be explained more clearly. For respondents who had not yet experienced sexual debut, the censoring time should correspond to the respondent’s age at interview. | Thank you for the comment. Further clarification has been provided in the methods section. | **Line 139 – Line 146** |
| 11. Methods – Age variable: The inclusion of current age group (15–19 vs 20–24) as an explanatory variable requires justification because age at first sexual intercourse is already used as the time variable in the survival model. | Thank you for this important observation. We acknowledge that age is used as the time scale in the survival analysis. However, the inclusion of current age group (15–19 vs 20–24) as an explanatory variable was intended to account for differences in exposure time among respondents. Therefore, the age group variable captures variations in risk profiles and timing patterns across different age groups. We have clarified this justification in the revised manuscript. | **Line 153 – Line 155** |
| 12. Results – Education interpretation: The Kaplan–Meier interpretation of education appears inconsistent with the results. The tables indicate that higher education is associated with delayed sexual debut, whereas the narrative suggests the opposite. | Thank you for the observation. We have made changes to the Kaplan Meier as we intepreted in reverse. | **Figure 2 and Table 3** |
| 13. Results – Marital status: The interpretation of marital status requires caution because sexual debut often occurs prior to marriage. Current marital status may therefore reflect reverse temporality. | Thank you for your observation. We have made changes to the interpretation. | **Line 274 – Line 276** |
| 14. Reporting p-values as “<0.05” limits the information available to readers. Providing the exact p-value (e.g., p = 0.032) improves transparency and allows readers to better assess the strength of statistical evidence. In addition, the p-value should correspond to the overall comparison across categories and should therefore be presented clearly in the p-value column associated with the variable. | Thank you for the observation. The exact p values have been insertedd in all the tables. | **Table 2, Table 3 and Table 4** |
| 15. Results – Hazard ratio interpretation: The narrative should refer to the hazard of earlier sexual debut rather than simply “hazard of sexual debut.” | Thank you for the observation. The suggested changes have been made in the results section. | **Whole results section** |
| 16. Results – Terminology consistency: The manuscript alternates between “young females,” “young women,” and “females.” It would be preferable to consistently use the term “young women.” | Thank you for the obersatio. The suggested use of young women has been adipted through out the manuscript. | **The whole manuscript** |
| 17. Results – p-value formatting: The log-rank p-value for phone ownership is reported as <0.000, which should instead be written as <0.001. | The exact p values have been inserted in the variable phone ownership. | **Table 3** |
| 18. Results – Small subgroup caution: Estimates for the “Other” religion category should be interpreted cautiously due to the very small sample size. | Thank you for the comment. This has been effected. | **Line 340 – Line 341** |
| 19. Statistical model assumptions: The Cox proportional hazards model assumes proportional hazards, meaning hazard ratios remain constant over time. While the manuscript explains this assumption theoretically, it does not report whether it was tested. The authors should indicate whether diagnostics such as Schoenfeld residual tests or log–log survival plots were conducted. | Thank you for the comment we have indicated the diagnostic tested used in the study. | **Line 197 – Line 204** |
| 20. Discussion – Study population: The opening sentence incorrectly states that the study included women aged 20–24 years. The analysis actually includes women aged 15–24 years. | The edit has been effected | **Line 285** |
| 21. Discussion – Age interpretation: The discussion should reconsider interpreting age group as a determinant of sexual debut given the time-scale structure of the survival model. | Thank you for the comment. The age interpretation has been revised. | **Line 299 – Line 300** |
| 22. Conclusion: The conclusion provides a useful summary but should ensure that only predictors significant in the multivariable model are emphasized and causal language is avoided. | Thank you for the comment. The conclusion paragraph has revised. | **Whole conclusion** |
| 23. References: Reference 27 appears to duplicate Reference 10 and should be removed. The remaining references should be renumbered accordingly. | The duplicated referneces have been removed. | **Reference section** |
| **Reviewer 4** | | |
| Reviewer #4: In this study, the authors used time to an event survival analysis, and the event of interest was sexual debut, that is, those who were sexually active, were coded 1, and those not sexually active were coded zero. This means, the survival analysis will model not sexually active, the authors are misinterpreting this to mean sexually active. Further, Figure 1, the survival curves are crossing, we can literally not see any differences, but the authors claim a difference which is wrongly interpreted anyway. | We are grateful for your favourable  comments and remarks. We would like to  express our appreciation for your valuable  expertise and the time you dedicated to our  work.  The survival figure have been correctly interpreted and we alsomade reference to the study conducted in Rwanda  “Kubahoniyesu, T., Namalinzi, F., 2024. Time to First Sexual Experience and Its Determinants Among Female Youths: A Survival Analysis of Rwanda Demographic Health Survey (2019-2020). Int. J. Womens Health Volume 16, 2011–2023. https://doi.org/10.2147/IJWH.S482932 | **Figure 2, Figure, 3, Fugure 4, and Figure 5** |
| Regarding Figure three, the authors say, “Females with higher education are more likely to initiate sexual activity earlier than those with no education (Figure 3)”. This is wrong, it is because of misunderstanding of the survival curves, it is actually the opposite. In fact, all graphs are wrongly interpreted, the notation of the formula on page 8 leaves much to be desired. Whereas the interpretation of the Cox model is correct, however, the assumptions of the Cox model as seen by most survival graphs are not satisfied, the authors should have used either a stratified Cox model or any other parametric survival model that do not assume constant hazard over time. | Thank you for the observation. The survival curves have now been correctly interpreted and the relevant sections revised accordingly. We also assessed the proportional hazards assumption and found only moderate violations. As supported by Peter C. Austin and Danilo Giardiello (2025), moderate violations of the proportional hazards assumption may have minimal impact on model calibration. Therefore, the Cox proportional hazards model remains appropriate for this analysis. | **Figure 2, Figure, 3, Fugure 4, and Figure 5** |
