# Supplementary material for: Time to first sexual experience and its predictors among young women (15–24 years) in Zambia: Evidence from 2024 Demographic and Health Survey
Source: PLOS Glob Public Health. 2026 Jun 22;6(6):e0006687. doi: 10.1371/journal.pgph.0006687 (PMC13286187; doi:10.1371/journal.pgph.0006687)
Supplement: Peer review history

# Peer review: Decision Letter 0

**DOI:** 10.1371/journal.pgph.0006687.r001

**Authors:** Nebechukwu Ugwu

24 Mar 2026

PGPH-D-26-00559

Time to first sexual debut and its predictors among young women (15-24 years) in Zambia: evidence from 2024 Zambia Demographic and Health Survey

PLOS Global Public Health

Dear Dr Emmanuel Musonda

Thank you for submitting your manuscript to PLOS Global Public Health. After careful consideration, we feel that it has merit but does not fully meet PLOS Global Public Health’s publication criteria as it currently stands. Therefore, we invite you to submit a revised version of the manuscript that addresses the points raised during the review process.

Please submit your revised manuscript by April 10, 2026. If you will need more time than this to complete your revisions, please reply to this message or contact the journal office at globalpubhealth@plos.org. When you're ready to submit your revision, log on to https://www.editorialmanager.com/pgph/ and select the 'Submissions Needing Revision' folder to locate your manuscript file.

Please include the following items when submitting your revised manuscript:

- A letter that responds to each point raised by the editor and reviewer(s). You should upload this letter as a separate file labeled 'Response to Reviewers'.
- A marked-up copy of your manuscript that highlights changes made to the original version. You should upload this as a separate file labeled 'Revised Manuscript with Track Changes'.
- An unmarked version of your revised paper without tracked changes. You should upload this as a separate file labeled 'Manuscript'.

Guidelines for resubmitting your figure files are available below the reviewer comments at the end of this letter.

We look forward to receiving your revised manuscript.

Kind regards,

Nebechukwu Henry Ugwu, Ph.D

Guest Editor

PLOS Global Public Health

Journal Requirements:

1. Please amend your online Financial Disclosure statement. If you did not receive any funding for this study, please simply state: "The authors received no specific funding for this work."

2. Please update your online Competing Interests statement. If you have no competing interests to declare, please state: "The authors have declared that no competing interests exist."

3. Please provide separate main figure files in .tif or .eps format only and ensure that all files are under our size limit of 10MB.

For more information about how to convert your figure files please see our guidelines: https://journals.plos.org/globalpublichealth/s/figures

4. If the reviewer comments include a recommendation to cite specific previously published works, please review and evaluate these publications to determine whether they are relevant and should be cited. There is no requirement to cite these works unless the editor has indicated otherwise.

Additional Editor Comments (if provided):

[Note: HTML markup is below. Please do not edit.]

Reviewers' comments:

Reviewer's Responses to Questions

**Comments to the Author**

1. Does this manuscript meet PLOS Global Public Health’s publication criteria? Is the manuscript technically sound, and do the data support the conclusions? The manuscript must describe methodologically and ethically rigorous research with conclusions that are appropriately drawn based on the data presented.

Reviewer #1: Yes

Reviewer #2: Partly

Reviewer #3: Yes

Reviewer #4: No

**********

2. Has the statistical analysis been performed appropriately and rigorously?

Reviewer #1: Yes

Reviewer #2: I don't know

Reviewer #3: No

Reviewer #4: No

**********

3. Have the authors made all data underlying the findings in their manuscript fully available (please refer to the Data Availability Statement at the start of the manuscript PDF file)?

The PLOS Data policy requires authors to make all data underlying the findings described in their manuscript fully available without restriction, with rare exception. The data should be provided as part of the manuscript or its supporting information, or deposited to a public repository. For example, in addition to summary statistics, the data points behind means, medians and variance measures should be available. If there are restrictions on publicly sharing data—e.g. participant privacy or use of data from a third party—those must be specified.

Reviewer #1: Yes

Reviewer #2: Yes

Reviewer #3: Yes

Reviewer #4: Yes

**********

4. Is the manuscript presented in an intelligible fashion and written in standard English?

PLOS Global Public Health does not copyedit accepted manuscripts, so the language in submitted articles must be clear, correct, and unambiguous. Any typographical or grammatical errors should be corrected at revision, so please note any specific errors here.

Reviewer #1: Yes

Reviewer #2: No

Reviewer #3: Yes

Reviewer #4: No

**********

5. Review Comments to the Author

Please use the space provided to explain your answers to the questions above. You may also include additional comments for the author, including concerns about dual publication, research ethics, or publication ethics. (Please upload your review as an attachment if it exceeds 20,000 characters)

Reviewer #1: This manuscript examines the timing of first sexual debut among young women aged 15–24 in Zambia using data from the 2024 Zambia Demographic and Health Survey (ZDHS). The authors employ Kaplan–Meier survival analysis, log-rank tests, and Cox proportional hazards regression to identify determinants of sexual debut. The topic is relevant to sexual and reproductive health (SRH), adolescent health policy, and HIV/STI prevention in sub-Saharan Africa. Overall, the manuscript has potential but requires improvement in certain areas.

1. The manuscript does not present a conceptual or theoretical framework guiding the analysis of sexual debut. Sexual debut is widely studied using frameworks such as Social Ecological Model, Health Belief Model, Life Course Perspective, etc. Without a guiding framework, the selection of variables appears largely descriptive. The authors should introduce a conceptual model explaining how socioeconomic, demographic and informational factors influence sexual initiation. This will also strengthen the Introduction as it is current somewhat brief.

2. Although the introduction provides statistics on STIs and teenage pregnancy, it does not clearly articulate what specific knowledge gap this study fills, why Zambia specifically requires further investigation and how this study advances existing research.

3. again, even though the authors applied Kaplan–Meier and Cox regression, the justification for survival analysis is not adequately explained. Why is survival analysis appropriate for DHS data? Was age at interview used as censoring time?

4. some interpretations in the results section appears problematic. For example, the authors state that “Females aged 20–24 had reduced hazards of sexual debut compared to those aged 15–19. However, this interpretation may reflect age cohort effects rather than causal relationships as the authors assumes. Similarly, the interpretation of marital status as a predictor of sexual debut is conceptually questionable because sexual debut often precedes marriage. The authors should reconsider how these variables are interpreted.

5. The discussion is largely descriptive and simply repeats results with limited analytical depth. A stronger discussion should explain why these associations exist and link findings to existing literature.

6. finally, the authors should read through for grammar and typographical errors. references 10 and 27 appear duplicated. The phrase “young females” could be replaced with “young women” for consistency with global health literature.

Reviewer #2: The authors have carried out a research on an issue of public health significance and have used the appropriate data analysis technique. There are however some concerns noted which have been highlighted below:

Comment 1. Line 23-27: “The results of the multivariable Cox regression analysis for determinants of time to first sexual experience among young females. Established that Determinants with reduced hazards ratio of timing to first sexual debut included females aged 20–24 years (AHR = 0.75, 95% CI: 0.70–0.80), Females from rich households (AHR26 = 0.75, 95% CI: 0.67–0.85), literate women (AHR = 0.87, 95% CI: 0.80–0.94)”

These two sentences should be merged and shortened

Comment 2. Line 38. “Sexual debut timing among young women continues to attract significant interest, as it marks the onset of exposure to various sexual and reproductive health outcomes”

This is grammatically wrong. Instead state that “it marks the onset of exposure to adverse sexual and reproductive health outcomes”.

Comment 3. Line 52. “Age, Education, religion, place of residence, region”

Education should start with a small letter, not a capital letter

Comment 4. Line 57. “When factors are identified and comprehended, this will influence policymakers and healthcare provide to develop strategies”

Healthcare providers not provide

Comment 5. Include that you applied survey weights in the methods section

Comment 6. Line 176. “Females with higher education are more likely to initiate sexual activity earlier than those with no education (Figure 3)”

According to the Kaplan Meiyer curve, the reverse is the case. You have not interpreted the Kaplan Meiyer curve correctly

Comment 7. “Females who are not working have a higher probability of experiencing the event compared those who are employed (Figure 4).

According to the Kaplan Meiyer curve, the reverse is the case. You have not interpreted the Kaplan Meiyer curve correctly

Comment 8. Line 178. Similarly, never-married females have a higher probability of first sexual experience occurring earlier than their married counterparts (Figure 5).

According to the Kaplan Meiyer curve, the reverse is the case. You have not interpreted the Kaplan Meiyer curve correctly

Comment 9. Line 196. There appears to be an inconsistency between the Kaplan–Meier survival curves and the bivariate Cox regression results for marital status. Since both analyses are unadjusted comparisons, they would generally be expected to show a similar direction of association. The authors are encouraged to verify the coding of the event variable, the reference category used in the Cox model, and the specification of the survival time variable. Clarification of this discrepancy would strengthen the validity of the analysis. This should be done for all the variables in the bivariate analysis.

Comment 10. Multivariable cox regression analysis is however in line with the Kaplan Meiyer curves

Comment 11. Line 220 “This study aimed to identify the factors associated with the time to first sexual experience among young females in Zambia aged 20–24 years.”

It should be 15 to 24 years.

Comment 12. “Therefore, there is urgent need for government to take keen interest in ensuring that schools starts to offer effective sexual programs which include comprehensive sexuality education program. Which can actually promote the delay in sexual initiations and enhancing the economic and social condition for young women”

These sentences should be merged but should not be too long.

Comment 13. Discussion: The discussion is very brief. Please give likely explanations for your findings e.g. on marital status, education, head of household. Other variables found significant but not discussed should be discussed e.g. use of mobile phones, literacy level.

Comment 14. Line 278. “Younger age, lower educational attainment, being from male-headed households, employment, lower wealth status, and being ever married were associated with a higher likelihood of early sexual initiation”

Comment 15. The conclusion states that being from male headed households was associated with higher likelihood of early sexual initiation. However, the multivariate Cox model states the reverse.

Please go through all your tables, graphs, results section and discussion and ensure they are in line. Re-do any analysis that was not properly done, probably in the bivariate analysis.

Note that your discussion on determinants should be based on findings from the multivariate Cox proportional hazards model and not from the bivariate analysis.

Reviewer #3: This manuscript addresses an important public health issue by examining the timing of first sexual debut among young women in Zambia using nationally representative data from the 2024 Zambia Demographic and Health Survey. The study is timely and has strong potential to contribute to understanding factors that influence early sexual initiation and to inform sexual and reproductive health policies and interventions in Zambia. The use of survival analysis techniques such as Kaplan–Meier curves and Cox proportional hazards models is appropriate for analysing the timing of sexual debut. The authors are commended for undertaking a study with important policy implications. However, several areas of the manuscript require clarification, methodological refinement, and language editing in order to strengthen the scientific rigor and clarity of the paper. The comments below are provided to support improvement of the manuscript.

Title: The title is relevant and communicates the topic clearly; however, it could be improved for clarity and precision. The phrase “time to first sexual debut” is somewhat repetitive because the term debut already implies the first occurrence. A clearer formulation would be “age at sexual debut” or “time to first sexual intercourse.”

Abstract – Introduction: The sentence stating that “little is known about this area of research in Zambia” is vague. It would be more appropriate to clarify that limited recent evidence exists, particularly using nationally representative data from the 2024 Zambia Demographic and Health Survey.

Abstract – Methods: The phrase “with the level of significance of 0.05” is verbose and should be replaced with the standard statistical phrasing “Statistical significance was set at p < 0.05.”

Abstract – Conclusion: The conclusion is relevant but slightly overreaches beyond the results presented in the abstract. It would be preferable for the conclusion to align closely with the predictors actually reported in the

abstract.

Abstract – Keywords and formatting: The term “Key word” should be corrected to “Keywords,” and capitalization inconsistencies throughout the abstract should be addressed.

Introduction – Grammar: The statement “the median age for sexual debut was 16 year” should be corrected to “16 years.”

Introduction – Variable capitalization: Variables such as Age, Education, religion, and place of residence should be consistently written in lowercase when used as general descriptors.

Introduction – Terminology: The manuscript alternates between the terms “sexual debut,” “sexual experience,” and “first sexual intercourse.” A single consistent term should be used throughout the manuscript.

Methods – Outcome variable: The description of the outcome variable requires clarification. While the authors indicate that age at first sexual intercourse was used as the time-to-event variable, they also mention constructing a binary variable for sexual experience. Cox proportional hazards models require both a time variable and an event indicator. The authors should clearly specify the time variable and censoring indicator.

Methods – Censoring: The handling of censored observations should be explained more clearly. For respondents who had not yet experienced sexual debut, the censoring time should correspond to the respondent’s age at interview.

Methods – Age variable: The inclusion of current age group (15–19 vs 20–24) as an explanatory variable requires justification because age at first sexual intercourse is already used as the time variable in the survival model.

Results – Education interpretation: The Kaplan–Meier interpretation of education appears inconsistent with the results. The tables indicate that higher education is associated with delayed sexual debut, whereas the narrative suggests the opposite.

Results – Marital status: The interpretation of marital status requires caution because sexual debut often occurs prior to marriage. Current marital status may therefore reflect reverse temporality.

Reporting p-values as “<0.05” limits the information available to readers. Providing the exact p-value (e.g., p = 0.032) improves transparency and allows readers to better assess the strength of statistical evidence. In addition, the p-value should correspond to the overall comparison across categories and should therefore be presented clearly in the p-value column associated with the variable.

Results – Hazard ratio interpretation: The narrative should refer to the hazard of earlier sexual debut rather than simply “hazard of sexual debut.”

Results – Terminology consistency: The manuscript alternates between “young females,” “young women,” and “females.” It would be preferable to consistently use the term “young women.”

Results – p-value formatting: The log-rank p-value for phone ownership is reported as <0.000, which should instead be written as <0.001.

Results – Small subgroup caution: Estimates for the “Other” religion category should be interpreted cautiously due to the very small sample size.

Statistical model assumptions: The Cox proportional hazards model assumes proportional hazards, meaning hazard ratios remain constant over time. While the manuscript explains this assumption theoretically, it does not report whether it was tested. The authors should indicate whether diagnostics such as Schoenfeld residual tests or log–log survival plots were conducted.

Discussion – Study population: The opening sentence incorrectly states that the study included women aged 20–24 years. The analysis actually includes women aged 15–24 years.

Discussion – Age interpretation: The discussion should reconsider interpreting age group as a determinant of sexual debut given the time-scale structure of the survival model.

Conclusion: The conclusion provides a useful summary but should ensure that only predictors significant in the multivariable model are emphasized and causal language is avoided.

References: Reference 27 appears to duplicate Reference 10 and should be removed. The remaining references should be renumbered accordingly.

Reviewer #4: In this study, the authors used time to an event survival analysis, and the event of interest was sexual debut, that is, those who were sexually active, were coded 1, and those not sexually active were coded zero. This means, the survival analysis will model not sexually active, the authors are misinterpreting this to mean sexually active. Further, Figure 1, the survival curves are crossing, we can literally not see any differences, but the authors claim a difference which is wrongly interpreted anyway.

Regarding Figure three, the authors say, “Females with higher education are more likely to initiate sexual activity earlier than those with no education (Figure 3)”. This is wrong, it is because of misunderstanding of the survival curves, it is actually the opposite. In fact, all graphs are wrongly interpreted, the notation of the formula on page 8 leaves much to be desired. Whereas the interpretation of the Cox model is correct, however, the assumptions of the Cox model as seen by most survival graphs are not satisfied, the authors should have used either a stratified Cox model or any other parametric survival model that do not assume constant hazard over time.

**********

6. PLOS authors have the option to publish the peer review history of their article (what does this mean?). If published, this will include your full peer review and any attached files.

**Do you want your identity to be public for this peer review?**  If you choose “no”, your identity will remain anonymous but your review may still be made public.

For information about this choice, including consent withdrawal, please see our Privacy Policy.

Reviewer #1: No

Reviewer #2: **Yes:**  Dr Tope Olubodun

Reviewer #3: **Yes:**  Samson Shumba

Reviewer #4: No

**********

[NOTE: If reviewer comments were submitted as an attachment file, they will be attached to this email and accessible via the submission site. Please log into your account, locate the manuscript record, and check for the action link "View Attachments". If this link does not appear, there are no attachment files.]

-->Figure Resubmissions:

While revising your submission, we strongly recommend that you use PLOS’s NAAS tool (https://ngplosjournals.pagemajik.ai/artanalysis) to test your figure files. NAAS can convert your figure files to the TIFF file type and meet basic requirements (such as print size, resolution), or provide you with a report on issues that do not meet our requirements and that NAAS cannot fix.

After uploading your figures to PLOS’s NAAS tool - https://ngplosjournals.pagemajik.ai/artanalysis, NAAS will process the files provided and display the results in the "Uploaded Files" section of the page as the processing is complete. If the uploaded figures meet our requirements (or NAAS is able to fix the files to meet our requirements), the figure will be marked as "fixed" above. If NAAS is unable to fix the files, a red "failed" label will appear above. When NAAS has confirmed that the figure files meet our requirements, please download the file via the download option, and include these NAAS processed figure files when submitting your revised manuscript.

Attachment Submitted filename: Time to first sexual debut.docx

Attachment Submitted filename: peer_review_report_sexual_debut_zambia.docx

# Author comment: Author response to Decision Letter 1

**DOI:** 10.1371/journal.pgph.0006687.r002

11 May 2026

Attachment Submitted filename: Rebuttal letter_final.docx

# Peer review: Decision Letter 1

**DOI:** 10.1371/journal.pgph.0006687.r003

**Authors:** Nebechukwu Ugwu, Nebechukwu Ugwu

2 Jun 2026

Time to first sexual experience and its predictors among young women (15-24 years) in Zambia: evidence from 2024 Demographic and Health Survey

PGPH-D-26-00559R1

Dear Dr Emmanuel Musonda

We are pleased to inform you that your manuscript 'Time to first sexual experience and its predictors among young women (15-24 years) in Zambia: evidence from 2024 Demographic and Health Survey' has been provisionally accepted for publication in PLOS Global Public Health.

Before your manuscript can be formally accepted you will need to complete some formatting changes, which you will receive in a follow up email. A member of our team will be in touch with a set of requests.

Please note that your manuscript will not be scheduled for publication until you have made the required changes, so a swift response is appreciated.

IMPORTANT: The editorial review process is now complete. PLOS will only permit corrections to spelling, formatting or significant scientific errors from this point onwards. Requests for major changes, or any which affect the scientific understanding of your work, will cause delays to the publication date of your manuscript.

If your institution or institutions have a press office, please notify them about your upcoming paper to help maximize its impact. If they'll be preparing press materials, please inform our press team as soon as possible -- no later than 48 hours after receiving the formal acceptance. Your manuscript will remain under strict press embargo until 2 pm Eastern Time on the date of publication. For more information, please contact globalpubhealth@plos.org.

Thank you again for supporting Open Access publishing; we are looking forward to publishing your work in PLOS Global Public Health.

Best regards,

Nebechukwu Henry Ugwu, Ph.D.

Guest Editor

PLOS Global Public Health

**********
